# Anteromedial Globus Pallidus Internus Deep Brain Stimulation for Gilles de la Tourette Syndrome: A Two-Case Report and Review of the Literature

**DOI:** 10.3390/neurolint18020021

**Published:** 2026-01-25

**Authors:** Tomislav Felbabić, Rok Berlot, Maja Trošt, Dejan Georgiev, Mitja Benedičič

**Affiliations:** 1 Department of Neurosurgery, University Medical Centre Ljubljana, 1000 Ljubljana, Slovenia; 2 Faculty of Medicine, University of Ljubljana, 1000 Ljubljana, Slovenia; 3 Department of Neurology, University Medical Centre Ljubljana, 1000 Ljubljana, Slovenia; 4 Artificial Intelligence Laboratory, Faculty of Computer and Information Science, University of Ljubljana, 1000 Ljubljana, Slovenia

**Keywords:** deep brain stimulation, Gilles de la Tourette syndrome, globus pallidus internus, anteromedial, case report

## Abstract

Background: Gilles de la Tourette syndrome is a neurobehavioral disorder that typically begins in childhood, subsides during puberty, and may reappear in adolescence. Treatment is primarily conservative, involving psychological and pharmacological therapy. Patients who do not respond to conservative therapy may be treated with deep brain stimulation, although this remains an experimental treatment. Methods: In this two-case report we present the first two cases of patients with Gilles de la Tourette syndrome in Slovenia treated with deep brain stimulation of the anteromedial globus pallidus internus. Results: Over an 18-month follow-up period, we observed an improvement in both cases. In the first case, the Yale Global Tic Severity Scale score decreased from 71 (17 for motor tics, 14 for phonic tics, and 40 on the impairment scale) to 44 points (12 motor, 12 phonic, and 20 impairment). In the second case, the score decreased from 72 (16 motor, 16 phonic, and 40 impairment) to 38 points (8 motor, 10 phonic, and 20 impairment). Conclusions: Deep brain stimulation could be a promising treatment for this disorder. However, further research is needed to determine the most suitable patients and targets.

## 1. Introduction

Gilles de la Tourette syndrome (GTS) is a neurobehavioral disorder characterized by involuntary motor and vocal tics [1]. It is estimated that up to 0.9% of children suffer from GTS [2]. It is characterized by a biphasic course. It typically occurs in childhood, then improves in adolescence and does not recur in most cases. However, in 25% of the patients, symptoms return in early adulthood and may even worsen over the years [3]. In such cases, treatment with behavioral therapy, drug therapy or a combination of both may be efficient [4]. For more resistant forms of the disease, deep brain stimulation (DBS) may be considered. Visser-Vandewalle et al. were the first to report a case of DBS for the treatment of GTS, in which the medial and intralaminar thalamic nuclei and the inner part of the ventral oral thalamic nuclei were stimulated bilaterally, in 1997 [5]. All three cases showed marked improvement with no serious complications. Since then, several papers have been published investigating the effectiveness of the stimulation of different nuclei in GTS. Despite these advances, DBS remains an experimental treatment option that should be used only in severely affected patients that are carefully selected and resistant to other treatments [6]. Case reports therefore provide valuable additional insights, and here we present the first two cases of DBS for the treatment of GTS from Slovenia, where we targeted the anteromedial part of the globus pallidus pars interna (amGPi).

## 2. Presentation of Cases

This manuscript is prepared according to CARE guidelines, which were added to the Supplementary Materials, Figure S3.

Case 1: In a 33-year-old male patient, mild motor tics began at the age of 4 years, followed by phonic tics. They were most pronounced between the ages of 10 and 15, then subsided until the age of 23 years, when they recurred. During childhood he was treated with risperidone and haloperidol until the age of 15. After relapse, he used diazepam 5 mg as needed. The tics manifested as a fast, dystonic twisting of the neck and abdominal muscles, gagging and coughing. On one occasion, he broke a rib due to a severe tic attack. At age 31, tetrabenazine was initiated and increased to 25 mg three times daily but discontinued after a few weeks due to nausea and vomiting. Psychiatric treatment included paroxetine 20 mg and mirtazapine 30 mg daily. Risperidone was reintroduced at 2 mg twice daily but stopped after 6 months because of weight gain, and haloperidol 0,5 mg twice daily was added. Antidepressants were later replaced with vortioxetine 10 mg daily. Due to further deterioration, zolpidem 10 mg as needed and biperiden 2 mg twice daily were added. Before the operation, the tics were in a waxing and waning pattern, but continuously disabling despite Habit Reversal Training, and he was unable to suppress them for more than 20 s. Given the severe, treatment-resistant course, DBS was proposed and accepted. Preoperatively, he was taking diazepam 5 mg as needed, zolpidem 10 mg as needed, biperiden 2 mg twice daily, haloperidol 0,5 mg twice daily, and vortioxetine 10 mg daily, along with antihypertensive therapy and asthma inhalations. His YGTSS score before surgery was 71 (motor 17, phonic 14, impairment 40).

Case 2: This 30-year-old male patient developed both motor and vocal tics at the age of 7, which remitted during puberty and recurred at 21. The tics presented as craniocervical dystonic movements and vocal tics without coprolalia. Symptoms were initially well controlled with amisulpride 300 mg twice daily and sertraline 100 mg daily, but worsened after 8 years. Amisulpride was discontinued and olanzapine 5 mg daily and clonazepam 0.4 mg three times daily were introduced. Botulinum toxin therapy was attempted, but provided only one week of benefit. Despite treatment changes, symptoms progressed. Behavioral therapy was ineffective. Tetrabenazine 25 mg three times daily was stopped after a few weeks because of fatigue and depression. It was replaced with biperiden 4 mg three times daily. Clonazepam was titrated to 3 mg three times daily, and vortioxetine 10 mg daily was added. After six months, amisulpride was reintroduced to the therapy at a dose of 100 mg twice daily, while sertraline and olanzapine were discontinued. Due to the severe, treatment-resistant course, the patient agreed to DBS. Preoperatively, he was receiving biperiden 4 mg and clonazepam 3 mg three times daily, vortioxetine 10 mg daily, and amisulpride 100 mg twice daily, plus aspirin and a beta-blocker after childhood surgery for aortic coarctation. His preoperative YGTSS score was 72 (motor 16, phonic 16, impairment 40).

## 3. Planning and Surgery

**Figure 1:**
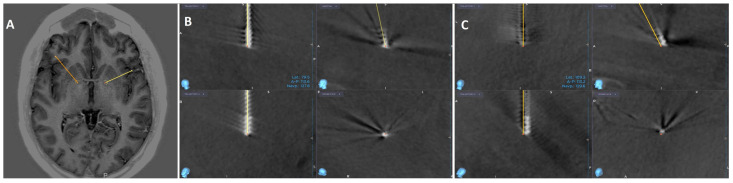
(**A**) Inverse recovery axial MR image of the planned targets for the first patient. (**B**,**C**) show the CT image after final lead placement for right (yellow) and left (orange) electrode, respectively, merged with the preoperative plan to confirm the final position. It is displayed in four different views: top left shows Trajectory 1, bottom left shows Trajectory 2, top right is the sagittal view, and bottom right is the probe’s eye view.

**Figure 2:**
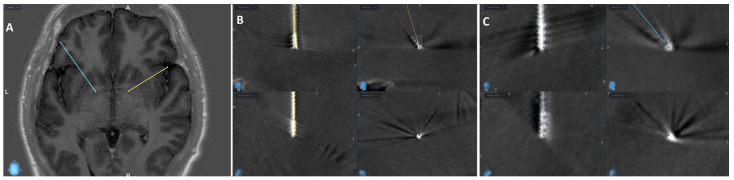
(**A**) Inverse recovery axial MR image of the planned targets for the second patient. (**B**,**C**) show the CT image after final lead placement for right (yellow) and left (blue) electrode, respectively, merged with the preoperative plan to confirm the final position. It is displayed in four different views: top left shows Trajectory 1, bottom left shows Trajectory 2, top right is the sagittal view, and bottom right is the probe’s eye view.

As part of the preoperative preparation, both patients underwent MRI of the head and CTA of cerebral vessels. The images were merged at a planning station (Medtronic S8; Medtronic, Minneapolis, MN, USA) and an operative plan was created. Axial MRI sequences with inversion recovery were used for targeting, with amGPi chosen bilaterally. The target was chosen based on imaging landmarks. In reference to the anterior/posterior commissure (AC/PC), the target was approximately 8 mm anterior to the midcommissural point, 14 mm lateral to the midline and 1 mm below the level of AC/PC plane in both cases. Under general anesthesia, a Leksell stereotactic ring was fixed and intraoperative head CT with O-arm (O-arm™ Surgical Imaging System; Medtronic, Minneapolis, MN, USA) was performed. The images were fused with the preoperative plan to determine stereotactic coordinates (Figure 1 and Figure 2).

Microelectrode recording (MER) guided the optimal placement of permanent electrodes (Medtronic 3389). During both surgeries we used 4 MER trajectories: central, anterior, medial and lateral. The central trajectory was selected bilaterally based on the absence of stimulation-induced side effects and concordance with preoperative imaging. Because the electrophysiological recording pattern from all four trajectories in both patients was inconsistent and presented as irregular, short-lasting, high-frequency activity, it was not used to select optimal trajectory [7]. After the electrodes were placed, we repeated intraoperative CT. Images were merged with the preoperative plan and in both cases the final electrode position was within 1 mm of the planned targets bilaterally. In the second phase, electrodes were connected to a neurostimulator (Percept PC B35200) via extension cables, implanted subcutaneously under the right clavicle. Both surgeries were uneventful, and patients awoke without neurological deficits. Below are the figures of the preoperative planned targets and final lead positions for both patients.

## 4. Follow-Up

On the first day after surgery, both patients reported a mild improvement, most likely due to the lesion or placebo effect, as the stimulation was still OFF. Both patients were discharged to home care 2 days after surgery. Therapy remained unchanged. Both patients had their systems activated two weeks after surgery and attended their first check-up two weeks after activation. They were then monitored at the neurology outpatient clinic at various intervals. Below is a summary of the postoperative course of both cases.

First case: Stimulation parameters were titrated with gradual increases in current, and interleaving stimulation was ultimately employed. No stimulation-related side effects were observed. Tic improvement allowed a stepwise tapering and eventual discontinuation of haloperidol, diazepam, zolpidem and biperiden. One month after activation, vortioxetine was replaced with sertraline, which the patient still takes as required for the mood disorder, along with added duloxetine and mirtazapine. He reported improved cognitive functioning. He was able to return to work. Eighteen months after surgery, he had an improved total score of 44 on the YGTSS (12 for motor, 12 for phonic tics and 20 on impairment scale). YGTSS was administered by the same neurologist both pre- and postoperatively.

Second case: Following the initial programming, the stimulation parameters were gradually titrated up until the patient no longer required further changes. Eventually, double monopolar stimulation demonstrated the best cost–benefit ratio. No specific side effects of stimulation have been recorded. He stopped taking vortioxetine 2 months after activation and amisulpride 12 months after activation, but continued therapy with biperiden and clonazepam, albeit in reduced doses, along with newly prescribed quetiapine 12 months after activation. One year after surgery, he found a job and subjectively reported a general improvement in quality of life as well as improvement in social interactions. Similarly to the first case, his YGTSS score improved: 18 months after surgery, the total YGTSS score was 38 (8 for motor tics, 10 for phonic tics, and 20 on the impairment scale). YGTSS assessment was performed by the same neurologist before and after surgery.

To facilitate a systematic review of the treatment course, changes in the clinical picture, and, based on these, adjustments to stimulation parameters and medication, we present the postoperative course of both patients in tabular form, which we added to the Supplementary Materials (Tables S1 and S2).

## 5. Discussion

We observed an improvement in GTS symptoms in both of our patients after amGPi DBS. The first patient’s YGTSS score improved from 71 points preoperatively to 44 points (38% improvement) 18 months after surgery. The second patient scored 72 points preoperatively and 38 points 18 months after surgery (47% improvement). In addition to tic reduction, the patients experienced benefits from a decreased oral medication burden and enhanced social functioning. During the follow-up neither patient experienced hardware-related issues such as infections, lead problems or other malfunctions. Due to the high stimulation parameters, the internal pulse generators (IPGs) of both patients were almost depleted after approximately one year. Consequently, both patients had a planned IPG replacement surgery, where we implanted rechargeable neurostimulators 12 months after system activation (Percept RC for the first patient and Activa RC for the second patient). Neither case had specific stimulation-related side effects (dysarthria, paresthesia, spasms).

A few studies have already reported on the effectiveness of GPi-DBS in GTS. The long-term outcome of GPi stimulation in the treatment of GTS was first described in 2005 [8]. In this study, the posterolateral part of the GPi (plGPi) was stimulated. They observed an improvement on the YGTSS scale of almost 50% after surgery. The plGPi represents the motor part of the nucleus and is generally a target for the treatment of hyperkinetic neurological disorders such as dystonia and dyskinesias in cognitively affected Parkinson’s disease [9,10]. The amGPi represents the associative/limbic part of the nucleus [11]. One of the hypotheses for the pathology of GTS is a disturbed associative/limbic connection in the basal ganglia [12]. For this reason, stimulation of amGPi has begun to be compared with other previously tested targets in the treatment of GTS.

The first double-blind report comparing the stimulation of the centromedian–parafascicular thalamic nucleus (CM-pf) to amGPi was conducted in 2008 [13]. The CM-Pf and the amGPi were stimulated bilaterally in three patients. The effects of bilateral CM-pf stimulation, bilateral amGPi stimulation, simultaneous stimulation of the CM-pf and amGPi, and no stimulation at 2-month intervals were compared in a double-blind fashion. They found that bilateral amGPi stimulation achieved the best effect with a 65%, 96% and 74% reduction in YGTSS in the first, second and third patients, respectively. Martinez-Fernandez et al. compared amGPi and plGPi stimulation and also described a better result with amGPi stimulation [14]. AmGPi was stimulated in two patients, plGPi in two patients, and plGPi first and after 18 months amGPi in one patient. The median percentage improvement in YGTSS after amGPi stimulation was 38% compared to 20% after plGPi stimulation.

There are several single-case reports on amGPi stimulation that describe an improvement compared to the preoperative state. Huasen et al. describe a case of emergency DBS in a GTS patient who had severe cervical myelopathy and tetrasymptomatics due to severe cervical motor tics in the form of violent extensions of the neck [15]. Cervical spine surgery would not have been safe due to the marked cervical motor tics, so they opted for amGPi DBS. They reported a 55% improvement in YGTSS 12 months after surgery. Dwarakanath et al. describe the first case of DBS treatment of GTS in India in which amGPi was stimulated [16]. Nine months after surgery, they report an improvement in YGTSS of more than 72%. In 2022, Srinivas et al. published a case of amGPi stimulation in a patient with GTS in which a 75% improvement in YGTSS was achieved within 6 months [17]. Similarly to us, Doshi et al. also published a double case report of amGPi stimulation in two patients with GTS [18]. After 18 months, they describe an improvement in YGTSS of 73% in the first case and 56% in the second case.

A series of GTS patients treated with amGPi DBS have already been described in some centers. Smeets et al. evaluated the outcome of five patients and observed a significant improvement in YGTSS postoperatively with a median improvement of 69% [19]. They conclude that amGPi stimulation is effective in the treatment of refractory GTS and possibly also obsessive–compulsive disorder (OCD) associated with GTS without serious complications or side effects. A similar conclusion was drawn by Azimi et al., who studied the outcome of amGPi stimulation in six patients with GTS [20]. They observed an average improvement of 63% after YGTSS after 1 year. The first double-blind study of a larger size was published in 2015 [21]. This double-blind study involved 15 patients with bilateral amGPi to treat GTS. Patients were then either stimulated or not stimulated for the first 3 months. After 3 months, the stimulated patients were turned off and the non-stimulated patients were turned on and re-examined after 3 months. The results of 13/15 patients were published and the median improvement was 8% OFF stimulation and 23% with ON stimulation. The comparison of the two stimulation periods shows a significant improvement during the ON period. However, three serious complications were also reported: two infections of the DBS material and one case of hypomania during the ON period. All three complications were successfully treated. The largest series of patients with amGPi stimulation to date was published in 2014 [22]. The same authors had already published a series of 11 patients [23], which was subsequently extended by a further six patients. The patients were followed up regularly—initially weekly for 1 month, then monthly for a total of 3 months and then every 3 months. The YGTSS was assessed 1 and 3 months postoperatively and then at the last visit 8 to 46 months postoperatively. There was a tendency for an improvement in tics over time, the median improvement being 43% at 1 month, 49% at 3 months postoperatively and 54% at the last visit, suggesting an improvement in progress in the first 3 months, followed by a plateau thereafter. Side effects of stimulation (anxiety, restlessness, dizziness, instability, and a worsening of pre-existing stuttering) were described in seven patients, most of which were temporary. Only one patient suffered a worsening of his tics and somatic symptoms with the stimulation and after 3 months opted to have it switched off permanently.

In addition to the YGTSS, there are other protocols for the assessment of GTS, such as the modified Rush Video-Based Tic Rating Scale, the Shapiro Tourette Syndrome Severity Scale, the Premonitory Urge for Tics Scale, the Tic Symptom Self-Report Scale, the Gilles de la Tourette Syndrome Quality of Life Questionnaire and others. In contrast to other protocols, only the YGTSS is used in all studies in which the DBS amGPi has been examined in the treatment of GTS. It is also the most widely used questionnaire and is recommended by the international GTS guideline. After a systematic literature review, it was found to be the most comprehensive, reliable and valid instrument for the assessment of tics [24]. Therefore, we only evaluated the YGTSS, as this allowed us to compare our results with all the studies listed above, where the reported range of improvement after amGPi stimulation ranges from 23% to 96%. In our two cases we observed 38% and 47% improvement, which is comparable with the literature.

Although both of our cases showed good results, the main limitation is the small sample size. Therefore, we cannot state with certainty that DBS of the amGPi nucleus in GTS has a positive effect, but only that it shows a potential benefit. We limited our outcome assessment to the YGTSS. Therefore, clinical domains beyond tic severity, including quality of life and psychiatric symptoms, could not be formally assessed. To maximize consistency, the YGTSS was administered by the same neurologist both pre- and postoperatively and under stable medication regimes. However, assessments were conducted using the patient’s usual stimulation settings, and no formal standardization, such as a blinded ON/OFF assessment, was undertaken. This would have allowed a more reliable distinction between the effects of DBS and potential placebo effects.

## 6. Conclusions

Our results support the growing body of evidence that amGPi DBS is a promising treatment for Tourette syndrome. However, further research and long-term follow-up are needed to optimize patient selection and stimulation parameters. Since GTS is a relatively rare indication for DBS, there is a need for large multicentric trials to further explore the efficacy of amGPi-DBS in comparison with other targets for the treatment of GTS.

## Data Availability

The dataset is available on request from the authors.

## Supplementary Materials

The following supporting information can be downloaded at: https://www.mdpi.com/article/10.3390/neurolint18020021/s1, Figure S1: Final lead position for case 1; Figure S2: Final lead position for case 2; Figure S3: CARE checklist; Table S1: Postoperative treatment course for case 1; Table S2: Postoperative treatment course for case 2.

## Abbreviations

The following abbreviations are used in this manuscript:

GTS	Gilles de la Tourette syndrome
DBS	Deep brain stimulation
YGTSS	Yale Global Tic Severity Scale
amGPi	Anteromedial globus pallidus internus
AC/PC	Anterior/posterior commissure
MER	Microelectrode recording
IPG	Internal pulse generator
plGPi	Posterolateral globus pallidus internus
CM-pf	Centromedian–parafascicular thalamic nucleus
OCD	Obsessive–compulsive disorder
