# Supplementary material for: Anteromedial Globus Pallidus Internus Deep Brain Stimulation for Gilles de la Tourette Syndrome: A Two-Case Report and Review of the Literature
Source: Neurol Int. 2026 Jan 25;18(2):21. doi: 10.3390/neurolint18020021 (PMC12943029; doi:10.3390/neurolint18020021)
Supplement: Supplementary file 1 [file neurolint-18-00021-s001.zip › Table 1.pdf]

	Clinical picture	Stimulation parameters	Medication
Before surgery	Fast, dystonic twisting of the neck and abdominal muscles, gagging and coughing.<br>YGTSS score 71 (motor 17, phonic 14, impairment 40).	/	diazepam 5 mg as needed<br>zolpidem 10 mg as needed<br>biperiden 2 mg twice daily<br>haloperidol 0,5 mg twice daily<br>vortioxetine 10 mg daily
After surgery (before activation)	Mild transient subjective improvement reported.	/	diazepam 5 mg as needed<br>zolpidem 10 mg as needed<br>biperiden 2 mg twice daily<br>haloperidol 0,5 mg twice daily<br>vortioxetine 10 mg daily
System activation (2 weeks after surgery)	Before activation same clinical picture as before the surgery.<br>After activation marked reduction in tic frequency, patient reported overall improvement in well-being.	Left GPi: 0-, 1-: 2 mA, 60 µs, 130 Hz; 2-: 3 mA, 60 µs, 130 Hz, Right GPi: 8-, 9-: 2 mA, 60 µs, 130 Hz; 10-: 3 mA, 60 µs, 130 Hz	diazepam 5 mg limited to twice daily<br>biperiden discontinued<br>zolpidem 10 mg as needed<br>haloperidol reduced to 0,3 mg twice daily<br>vortioxetine 10 mg daily
2 weeks after activation	Satisfied with tic frequency, but condition fluctuated considerably; when he was more agitated or anxious, there were more tics.<br>Reported improved memory and mental abilities.	Kept the previous stimulation parameters as programme B<br>Added programme A: left GPi: -1-: 3 mA, 60 µs, 130 Hz; right GPi: 8-, 9-: 3.0 mA, 60 µs, 130 Hz<br>Added programme C: left GPi: 2-: 4 mA, 60 µs, 130 Hz; right GPI: 10-: 4 mA, 60 µs, 130 Hz	diazepam discontinued<br>zolpidem 10 mg as needed<br>haloperidol discontinued<br>vortioxetine 10 mg daily
1 month after activation	Programme A: tics were not adequately controlled, though less severe than pre-operatively.<br>Programme B: adverse effects, including panic attacks and restlessness.<br>Programme C: best overall control. He also reported fatigue and anhedonia.	Adjusted programme A: left GPi: 2-, 4.0 mA, 3-: 3.0 mA, 60 µs, 130 Hz; right GPi: 10-, 4.0 mA, 11-: 3.0mA, 60 µs, 130 Hz<br>Adjusted programme B: left GPi: 1-, 3.0 mA; 2-: 4.0 mA, 60 µs, 130 Hz; right GPi: 9-, 3.0 mA; 10-: 4.0 mA, 60 µs, 130 Hz<br>Programme C unchanged	zolpidem 10 mg as needed<br>switch from vortioxetine to sertraline 50 mg daily
3 months after activation	An increase in tic frequency compared to the previous referral, including coughing, sniffing, blinking, shoulder shrugging, and additional vocal tics, as well as more anhedonia and fatigue.<br>Regarding tic control, programme B was the most effective, and there were no stimulation-related adverse effects.	Programme B unchanged<br>Adjusted programme A: left GPi: 2-, 5.2 mA, 60 µs, 180 Hz; right GPi: 10-, 5.2 mA, 60 µs, 180 Hz.<br>Adjusted programme C: Interleaving stimulation<br>Left GPi:, 1- and 2-: 4.5 mA, 60 µs, 125 Hz; right GPi: 9- and 10-: 4.5 mA, 60 µs, 125 Hz	zolpidem discontinued<br>Increased sertraline to 100 mg daily<br>Added quetiapine 25 mg in the evenings
4 months after activation	Tics were mild and reduced in frequency. Programme A suited him best. We recommend a gradual return to work on a half-day basis.	Programme A unchanged<br>Adjusted programme B: left GPi: 2-: 5.6 mA, 60 µs, 180 Hz; right GPi: 10-: 5.6 mA, 60 µs, 180 Hz	sertraline 100 mg daily<br>quetiapine 25 mg in the evenings

		Adjusted programme C:<br>Interleaving stimulation<br>Left GPI:, 1- and 2-: 5.2 mA, 60 μs, 125 Hz; right GPI: 9- and 10-: 5.2 mA, 60 μs, 125 Hz.	
6 months after activation	Condition remained stable with tic control considered satisfactory. Programmes A and B were tolerated well, whereas programme C was associated with discomfort. Stressful period was anticipated due to return to work and moving.	Programme A and B unchanged<br>Adjusted programme C: left GPi: 2-: 6.0 mA, 60 μs, 130 Hz; right GPi: 10-: 6.0 mA, 60 μs, 130 Hz	sertraline 100 mg daily<br>quetiapine 25 mg in the evenings<br>Added duloxetine 60 mg daily
9 months after activation	Tics were mild, significantly improved mood. He enjoyed going to work, he had 4 hours reduced working time, tics more frequent when experiencing fatigue. Programs A and B were equally effective for him, but program C was not suitable.	Programme A unchanged<br>Adjusted program B by setting double unipolar stimulation: Left GPi: 2-: 4.4 mA, 1-: 2.2 mA, 60 μs, 180 Hz; right GPi: 10-: 4.4 mA, 9-: 2.2 mA, 60 μs, 180 Hz<br>Programme C deleted	sertraline 100 mg daily<br>quetiapine 25 mg in the evenings<br>duloxetine 60 mg daily
12 months after activation	No notable differences between programs A and B. Occasional malaise and irritability, along with stress-related increases in tic frequency.	IPG switch from Percept PC to Percept RC<br>Program A unchanged<br>Adjusted program B: Left GPi: 2-: 4.4 mA, 3-: 2.2 mA, 60 μs, 180 Hz; right GPi: 10-: 4.4 mA, 11-: 2.2 mA, 60 μs, 180 Hz<br>Added programme C: Left GPi: 2-: 5.8 mA, 60 μs, 210 Hz; right GPi: 10-: 5.8 mA, 60 μs, 210 Hz	sertraline lowered to 50 mg daily<br>quetiapine discontinued<br>duloxetine 60 mg daily<br>Added mirtazapine 30 mg daily
18 months after activation	He reported an improvement in his condition. The tics were only mild, and his mood was better. Programme C suits him best, he also uses programme A occasionally, while programme B does not suit him. YGTSS score 44 (12 motor, 12 phonic, 20 impairment).	Programme A and C unchanged<br>Programme B deleted	sertraline 50 mg daily<br>duloxetine 60 mg daily<br>mirtazapine 30 mg daily

Table 1: Presentation of the postoperative treatment course for case 1. The »Clinical picture« column describes the patient's condition at the time of evaluation, based on which we decided on changes in stimulation programming and therapy. YGTSS: Yale global tic severity score, amGPi: anteromedial globus pallidus internus, IPG: internal pulse generator
