# Supplementary material for: Anteromedial Globus Pallidus Internus Deep Brain Stimulation for Gilles de la Tourette Syndrome: A Two-Case Report and Review of the Literature
Source: Neurol Int. 2026 Jan 25;18(2):21. doi: 10.3390/neurolint18020021 (PMC12943029; doi:10.3390/neurolint18020021)
Supplement: Supplementary file 1 [file neurolint-18-00021-s001.zip › Table 2.pdf]

	Clinical picture	Stimulation parameters	Medication
Before surgery	Craniocervical dystonic motor tics and vocal tics without coprolalia. YGTSS score 72 (motor 16, phonic 16, impairment 40).	/	biperiden 4 mg twice daily<br>clonazepam 3 mg three times daily<br>vortioxetine 10 mg daily<br>amisulpride 100 mg twice daily
After surgery (before activation)	Mild improvement reported, but no formal assesment.	/	biperiden 4 mg twice daily<br>clonazepam 3 mg three times daily<br>vortioxetine 10 mg daily<br>amisulpride 100 mg twice daily
System activation (2 weeks after surgery)	Before activation same clinical picture as before the surgery.<br>After activation only occasional tics remained, occurring mainly when he had to concentrate particularly hard.	Double monopolar stimulation:<br>Left GPI: 1-,2-, 5.4 mA; 40 µs; 150 Hz;<br>Right GPI: 9-,10-, 5.4 mA, 40 µs, 150 Hz	biperiden 4 mg twice daily<br>Reduced clonazepam to 2 mg three times daily<br>vortioxetine 10 mg daily<br>amisulpride 100 mg twice daily
2 weeks after activation	Stable condition, only isolated mild to moderate myoclonic tics were present, which worsened in stressful situations.	Double monopolar stimulation:<br>Left GPi: 1-,2-, 5.8 mA; 40 µs; 150 Hz;<br>Right GPi: 9-,10-, 5.8 mA; 40 µs; 150 Hz	biperiden 4 mg twice daily<br>clonazepam to 2 mg three times daily<br>vortioxetine 10 mg daily<br>amisulpride 100 mg twice daily
2 months after activation	Condition worsened slightly, with an increase in tic frequency. He also described a rapid flow of thoughts, rapid associations, high energy levels, and occasional sleep disturbances.	Adjusted previous parameters, which were saved as programme B:<br>Double monopolar stimulation:<br>Left GPi: 1-, 2-, 6 mA, 40 µs, 150 Hz;<br>Right GPi: 9-, 10-, 6 mA, 40 µs, 150 Hz<br>Added programme C:<br>Interleaving stimulation<br>Left GPi: 1- and 2-: 5.8 mA, 40 µs, 125 Hz;<br>Right GPi 9- and 10-: 5.8 mA, 40 µs, 125 Hz	biperiden 4 mg twice daily<br>clonazepam to 2 mg three times daily<br>vortioxetine discontinued<br>amisulpride 100 mg twice daily
3 months after activation	Felt worse, his tics intensified.	Programme B unchanged<br>Adjusted programme C:<br>Interleaving stimulation Left GPi: 1- and 2-: 6 mA, 40 µs, 150 Hz;<br>Right GPi 9- and 10-: 6 mA, 40 µs, 150 Hz	biperiden 4 mg twice daily<br>clonazepam to 2 mg three times daily<br>amisulpride 100 mg twice daily
6 months after activation	Condition improved, only isolated motor tics were present, with no vocal tics. He mainly used programme B, as programme C was less effective	Switched to programme B - see above.	biperiden reduced to 3 mg twice daily<br>clonazepam to 2 mg three times daily<br>amisulpride 100 mg twice daily
9 months after activation	Condition was improving, frequency of tics varied depending on the day. He found a job and was living a practically normal life.	Adjusted programme B:<br>Double monopolar stimulation:<br>Left GPi: 1-, 2-, 6.5 mA, 60 µs, 150 Hz; right GPi: 9-, 10-, 6.5 mA, 60 µs, 150 Hz	biperiden 3 mg twice daily<br>clonazepam to 2 mg three times daily<br>amisulpride 100 mg twice daily

12 months after activation	Slightly more vocal tics reported, but otherwise he had reintegrated socially into a practically normal life.	IPG switch from Percept PC (constant current stimulation) to Active RC (constant voltage stimulation)<br>Adjusted programme B: Double monopolar stimulation<br>Left GPi 1-, 2-, 7V, 60 µs, 150 Hz,<br>Right GPi: 9-,10-, 7 V, 60 µs, 150 Hz)	biperiden 3 mg twice daily<br>clonazepam to 2 mg three times daily<br>amisulpride discontinued<br>Added quetiapine 25 mg up to twice daily
18 months after activation	Condition was stable, only mild motor tics, no vocal tics. YGTSS score 38 (8 motor, 10 phonic, 20 impairment)	Programme B unchanged	biperiden 3 mg twice daily<br>clonazepam to 2 mg three times daily<br>quetiapine 25 mg up to twice daily

Table 2: Presentation of the postoperative treatment course for case 2. The »Clinical picture« column describes the patient's condition at the time of evaluation, based on which we decide on changes in stimulation programming and therapy. YGTSS: Yale global tic severity score, amGPi: anteromedial globus pallidus internus, IPG: internal pulse generator
