# Supplementary figures and images for: Anteromedial Globus Pallidus Internus Deep Brain Stimulation for Gilles de la Tourette Syndrome: A Two-Case Report and Review of the Literature
Source: Neurol Int. 2026 Jan 25;18(2):21. doi: 10.3390/neurolint18020021 (PMC12943029; doi:10.3390/neurolint18020021)

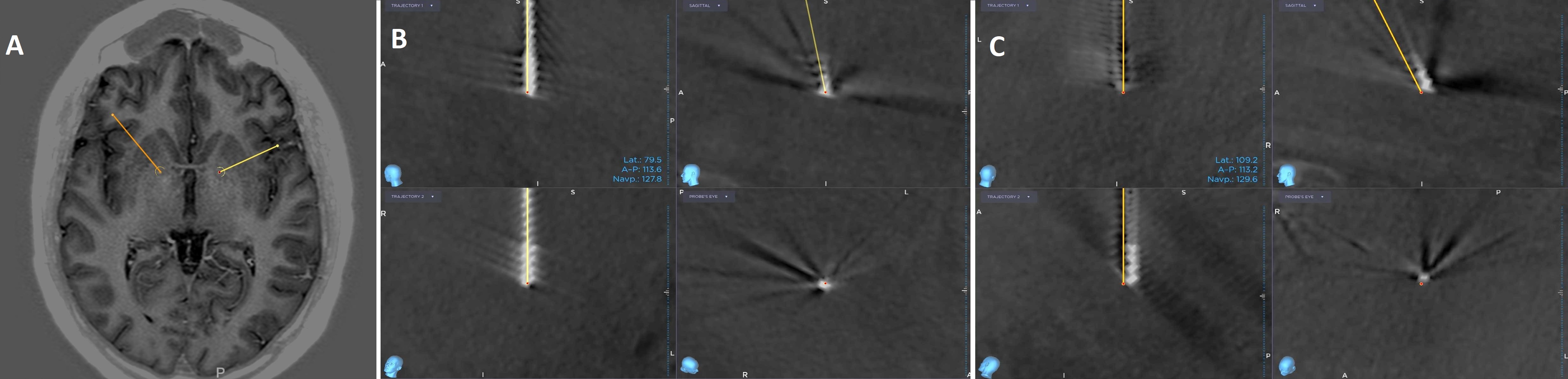

Supplement: Supplementary file 1 [file neurolint-18-00021-s001.zip › Figure 1.jpg]

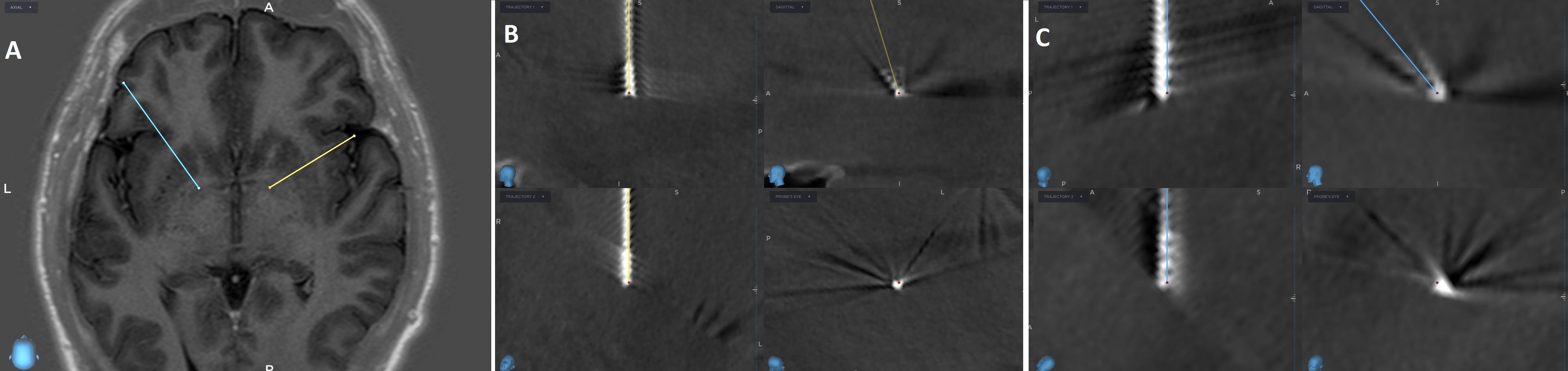

Supplement: Supplementary file 1 [file neurolint-18-00021-s001.zip › Figure 2.jpg]

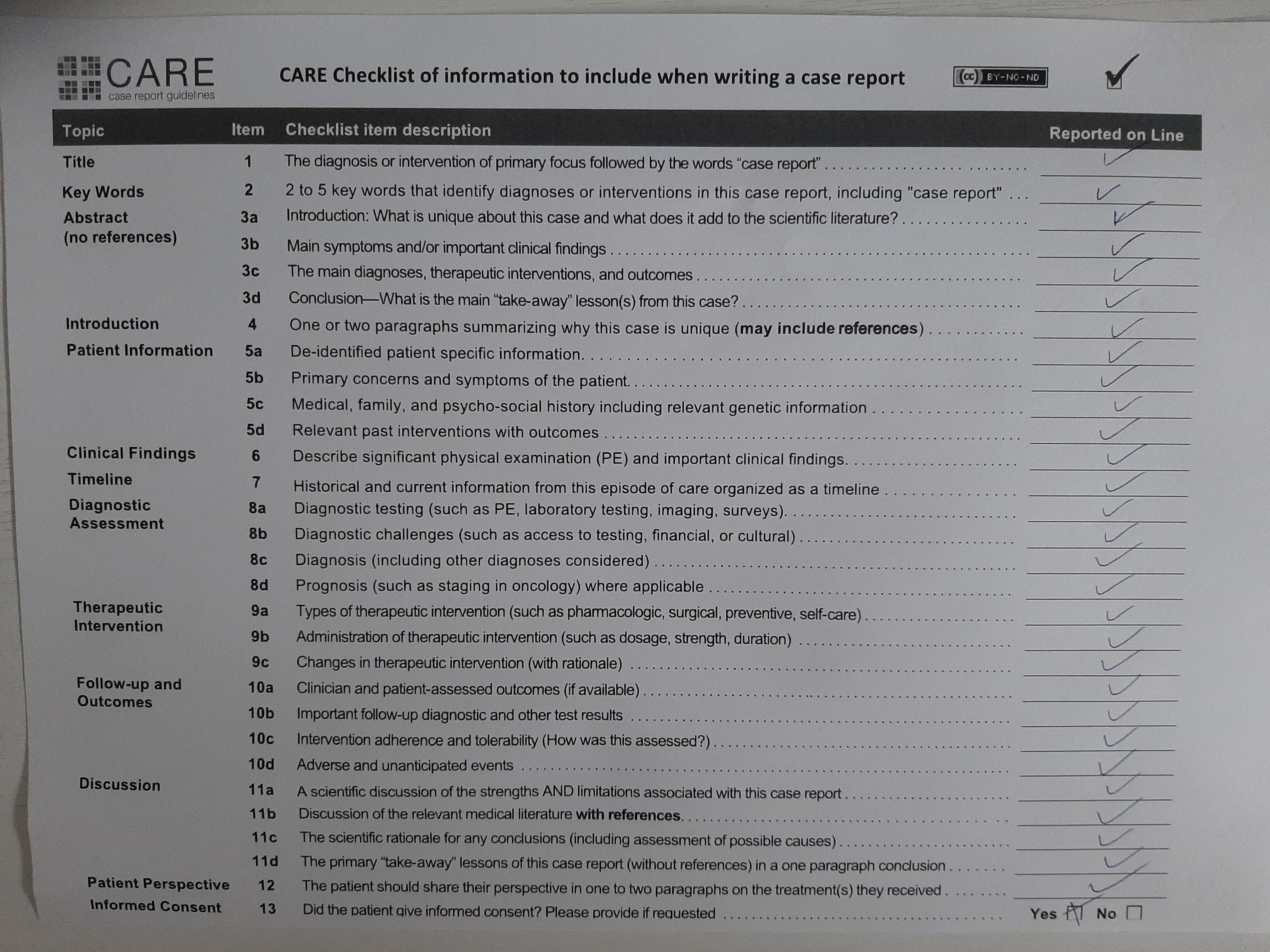

Supplement: Supplementary file 1 [file neurolint-18-00021-s001.zip › Figure 3.jpg]
